# EVIDENCE-BASED REVIEWS & RECOMMENDATIONS FROM THE BRAZILIAN SOCIETY OF SHOULDER AND ELBOW SURGERY: BIOINDUCTIVE IMPLANT AUGMENTATION VERSUS CONVENTIONAL REPAIR FOR ROTATOR CUFF TEARS

**DOI:** 10.1590/1413-785220263401e308954

**Published:** 2026-08-21

**Authors:** Paulo Henrique Schmidt Lara, Paulo Santoro Belangero, Márcio Schiefer, Leonardo Zanesco, Marcos Rassi Fernandes, Joel Murachovsky, João Felipe de Medeiros, Eduardo Angeli Malavolta

**Affiliations:** 1 Escola Paulista de Medicina, UNIFESP, Sao Paulo, SP, Brazil.; 2 Instituto Nacional de Traumatologia e Ortopedia Jamil Haddad (INTO), Rio de Janeiro, RJ, Brazil.; 3 Universidade de Sao Paulo, Faculdade de Medicina, Hospital das Clinicas HCFMUSP, Sao Paulo, SP, Brazil.; 4 Universidade Federal de Goias, Faculdade de Medicina, Goiania, GO, Brazil.; 5 Faculdade de Medicina do ABC, Santo Andre, SP, Brazil.; 6 Universidade Federal do Rio Grande do Norte, Natal, RN, Brazil.

**Keywords:** Rotator Cuff Injuries, Bioprosthesis, Tissue Scaffolds, Collagen, Arthroscopy, Lesões do Manguito Rotador, Bioprótese, Alicerces Teciduais, Colágeno, Artroscopia

## Abstract

**Introduction:**

Despite advances in arthroscopic techniques and tendon fixation methods, structural failure after rotator cuff repair remains a relevant clinical challenge, particularly in extensive and massive tears, poor tendon quality, and repairs performed under tension. In this context, tendon-bone healing is recognized as a key determinant of surgical success, driving interest in strategies to optimize the biological environment of the repair, such as bioinductive collagen implants (patches).

**Methods:**

A systematic review was conducted in PubMed, Embase, Cochrane Library, and the Virtual Health Library (VHL) up to March 2026, to evaluate whether rotator cuff repair augmented with a bioinductive implant (patch) is more effective than conventional rotator cuff repair for the treatment of rotator cuff tears. The certainty of evidence was assessed with the GRADE approach.

**Results:**

Four randomized controlled trials (288 participants) were included, with assessment of the re-tear rate and shoulder function. The results favored bioinductive implant augmentation, mainly through a lower re-tear rate and earlier functional recovery.

**Conclusion:**

To date, there is very low-quality evidence that rotator cuff repair augmented with a bioinductive implant (patch) is more effective than conventional repair for the treatment of rotator cuff tears (including complete and massive tears), with respect to the outcomes of re-tear and function. ***Level of evidence II; Systematic review.***

## RESUMO

**Introdução:**

Apesar dos avanços nas técnicas artroscópicas e nos métodos de fixação tendínea, a falha estrutural após o reparo do manguito rotador ainda é um desafio clínico relevante, especialmente em lesões extensas e maciças, em tendões de má qualidade e em reparos sob tensão. Nesse contexto, a cicatrização tendão-osso é reconhecida como um dos principais determinantes do sucesso cirúrgico, o que tem impulsionado o interesse por estratégias capazes de otimizar o ambiente biológico do reparo, como os implantes bioindutivos de colágeno (patches).

**Métodos:**

Realizou-se revisão sistemática da literatura nas bases PubMed, Embase, Cochrane Library e Biblioteca Virtual em Saúde (BVS) até março de 2026, para avaliar se o reparo do manguito rotador associado a reforço com implante bioindutivo (patch) é mais efetivo do que o reparo convencional para o tratamento das lesões do manguito rotador. A certeza da evidência foi avaliada pela abordagem GRADE.

**Resultados:**

Foram incluídos quatro ensaios clínicos randomizados (288 participantes), com avaliação da taxa de re-ruptura e da função do ombro. Os resultados favoreceram o reforço com implante bioindutivo, sobretudo por menor taxa de re-rruptura e recuperação funcional mais precoce.

**Conclusão:**

Até o momento, existe evidência de muito baixa qualidade de que o reparo do manguito rotador associado a reforço com implante bioindutivo (patch) é mais efetivo do que o reparo convencional para o tratamento das lesões do manguito rotador (incluindo lesões completas e maciças), em relação aos desfechos re-rruptura e função. ***Nível de evidência II; Revisão sistemática.***

## INTRODUCTION

Rotator cuff tears are among the most frequent causes of shoulder pain and dysfunction, with a substantial impact on functional capacity, quality of life, and occupational performance. Despite advances in arthroscopic techniques and tendon fixation methods, structural failure after rotator cuff repair remains a relevant clinical challenge, particularly in extensive and massive tears, poor tendon quality, and repairs performed under tension. In this context, tendon-bone healing has been recognized as one of the main determinants of surgical success, which has driven interest in strategies capable of optimizing the biological environment of the repair.^1–3^

Traditionally, surgical treatment of rotator cuff tears has focused on the mechanical restoration of tendon continuity through suturing and fixation to the footprint. However, evidence accumulated over recent decades shows that anatomical restoration alone is not always sufficient to ensure durable healing, especially in patients with advanced tendon degeneration, muscle atrophy, fatty infiltration, and/or previous surgery. The concept of biological augmentation has therefore gained prominence, aiming to promote tissue integration, improve repair quality, and reduce the incidence of re-tear.^2,4,5^

Among the currently available strategies, bioinductive collagen implants (patches) have received increasing attention as a promising alternative for rotator cuff repair. These devices, generally composed of a resorbable collagen matrix, are applied to the bursal surface of the tendon to act as a biological scaffold, stimulating the formation of new tendon tissue and contributing to the thickening and maturation of the repair. Unlike structural grafts used for mechanical supplementation, the bioinductive patch follows a predominantly biological approach aimed at modulating local healing.^3,5–7^

Although initial results have been encouraging, especially regarding the structural integrity of the repair and the increase in tendon thickness on imaging, uncertainties persist regarding the magnitude of the clinical benefit, the ideal indications, and the real impact on long-term functional outcomes. Moreover, the methodological heterogeneity of the published studies and the lack of standardization regarding the profile of treated lesions and patients hinder the consolidation of definitive recommendations for routine use.^6,7^ Given this context, this consensus was developed to critically analyze the role of bioinductive implants as an augmentation method in rotator cuff repair and to evaluate whether this technique is more effective than conventional repair for the treatment of rotator cuff tears.^6–8^

This study was conducted at the initiative of the Brazilian Society of Shoulder and Elbow Surgery with the participation of Cochrane Brazil.

### Research Question

Is rotator cuff repair augmented with a bioinductive implant (patch) more effective than conventional rotator cuff repair for the treatment of rotator cuff tears?

## METHODS

- Databases searched: PubMed, Embase, Cochrane Library, and the Virtual Health Library (VHL).
- Descriptors: As described in Appendix 1.
- Search period: Until March 2026.
- Study selection: Updated systematic reviews (SRs) with adequate methodological quality were prioritized. In the absence of suitable SRs, randomized controlled trials (RCTs) were considered, provided that their methodological quality had been assessed.

## RESULTS

The search retrieved 84 references (26 in PubMed, 41 in Embase, 17 in the Cochrane Library, and 0 in the Regional Portal BVS). After the initial assessment, 45 duplicate records were removed. Following abstract screening, 33 studies were excluded, leaving six studies for full-text evaluation. Two systematic reviews on the topic were assessed but not included.

**Figure 1:**
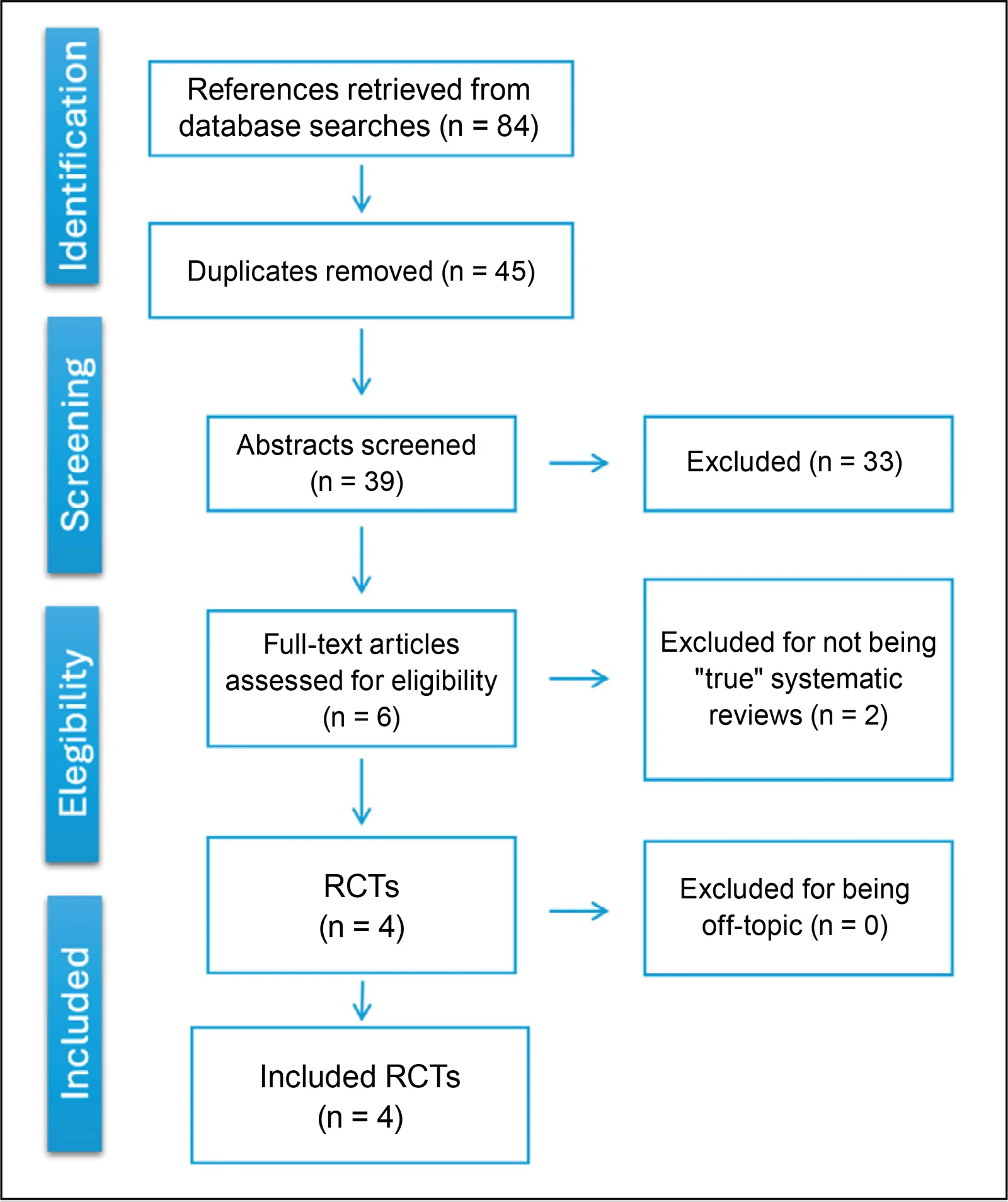
Flow diagram of the literature search.

Warren et al.,^2^ in a systematic review, included 13 studies (retrospective cohort studies, case series, and case reports) evaluating partial- and full-thickness rotator cuff tears. The outcomes assessed included the American Shoulder and Elbow Surgeons (ASES) score, the Constant-Murley score, the visual analog scale (VAS) for pain, the minimal clinically important difference, tendon thickness, complication and re-tear rates, and implant-related adverse events. The authors concluded that the bioinductive collagen patch appears to be a safe and promising strategy for augmenting rotator cuff repair—associated with significant clinical improvement, increased tendon thickness, and a possible reduction in the re-tear rate—while emphasizing that higher-quality comparative studies are still needed to define its role relative to conventional repair. This review was not included in the present consensus because it pooled several study types (retrospective cohorts, case series, and case reports). Longo et al.,^4^ in a systematic review, comprehensively reviewed the literature on bioinductive collagen implants in rotator cuff tears, including partial and complete tears, isolated implant use, use combined with repair, histological data, and healthcare costs, totaling 25 primary studies of various designs. The review reported overall clinical improvement, increased tendon thickness, reduced defect size, and histological findings consistent with favorable tissue remodeling, supporting the biological rationale of the implant. However, the heterogeneity of the studies and the predominance of observational evidence limit definitive conclusions about its clinical superiority over conventional repair. This review was also not included because it combined different types of primary studies (Figure 1).

### Included studies

No systematic review restricted to randomized controlled trials was found; therefore, four randomized controlled trials were included.

Barros et al.,^8^ in a randomized controlled trial, compared the clinical and radiological outcomes and re-tear rates of massive rotator cuff repair using a standard technique versus augmentation with a bioinductive collagen scaffold, with 12-month follow-up. Sixty-three patients were included: 29 in the augmented-repair (AR) group and 34 in the standard-repair (SR) group. In the AR group (median age 64 years), the mean Constant score (CS) improved from 49 preoperatively to 70, 86, and 89 at 3, 6, and 12 months, and the mean VAS improved from 7.5 to 2.9, 1.5, and 0.7. In the SR group (mean age 67 years), the mean CS improved from 52 to 62, 78, and 82, and the mean VAS from 7.2 to 4.5, 2.1, and 1.0. There were 2 re-tears (7%) and 2 cases of adhesive capsulitis in the AR group, versus 4 re-tears (13%) and 1 case of adhesive capsulitis in the SR group. The authors concluded that, at 12 months, augmented repair resulted in less pain, better functional scores, and a lower re-tear rate than standard repair, with the difference most evident at 3 and 6 months.

Ruiz Ibán et al.,^5^ in a randomized controlled trial, included 124 participants with complete posterosuperior rotator cuff tears, allocated to transosseous-equivalent repair alone (control) or with a bioinductive collagen patch applied over the repair (intervention). Of the 124 randomized patients (mean age 58.1 years), 114 (91.9%) underwent MRI at a mean of 25.4 months. The re-tear rate was lower in the intervention group (12.3%; 7 of 57) than in the control group (35.1%; 20 of 57) (P = 0.004; relative risk 0.35; 95% CI 0.16–0.76). Clinical scores improved in both groups (P < 0.001), with 87% achieving the minimal clinically important difference for the CMS and 90% for the ASES score, without significant between-group differences. The authors concluded that augmentation of a transosseous-equivalent repair in posterosuperior tears clearly reduces the re-tear rate at two years without increasing complications and with comparable clinical outcomes. Chacón et al.,^9^ in a prospective, double-blind, single-center randomized controlled trial, evaluated 60 patients with small to medium (≤ 2.5 cm) full-thickness supraspinatus tears, allocated to transosseous-equivalent arthroscopic repair (control, n = 30) or isolated bioinductive repair (intervention, n = 30). The primary outcome was tendon quality on biopsy at 6 months; secondary outcomes included patient-reported outcomes (ASES and CMS scores, VAS), tendon thickness and healing on MRI at 6, 12, and 24 months, satisfaction, and time to return to work. Histology at 6 months showed highly organized, parallel collagen bundles without inflammation in all intervention patients, versus poorly organized fibers in 24 of 30 (80%) controls (P < 0.0001). The increase in tendon thickness on MRI at 6 months was greater in the intervention group (2.0 mm vs 0.8 mm; P < 0.0001), which also showed higher ASES and CMS scores, less pain, greater satisfaction, and a faster return to work (median 90 vs 163.5 days; P < 0.0001). The authors concluded that, compared with conventional repair, bioinductive repair resulted in superior tendon quality, better patient outcomes, and earlier return to work.

Wang et al.,^10^ in a 2026 randomized controlled trial, compared conventional arthroscopic repair after completion of the tear with bioinductive collagen implant augmentation for high-grade partial-thickness supraspinatus tears. Forty-one patients (aged 35 to 75 years) refractory to conservative treatment were included. The primary outcome was the Western Ontario Rotator Cuff Index (WORC) score at 3 months; secondary outcomes included the ASES and Constant scores, return to activities, and tendon integrity on MRI. The implant group showed statistically superior early functional recovery, with better WORC scores at 6 weeks and 3 months and better ASES scores at 6 weeks, as well as earlier return to work and sport. This advantage was predominantly observed early, with no significant between-group differences at 6 and 12 months, and structural healing on MRI at 12 months was comparable between techniques. The authors concluded that bioinductive collagen implantation may accelerate early functional recovery in high-grade partial-thickness tears, without demonstrating sustained structural or functional superiority at one year.

The general characteristics of the included studies and the certainty of evidence according to the Grading of Recommendations Assessment, Development and Evaluation (GRADE) approach are presented in Tables 1 and 2, respectively.^11,12^

**Table 1:** Characteristics of the included studies.

Included study	Study design	Sample size	Lesion type / follow-up
Ferreira de Barros et al.^8^	RCT	63 (29 AR vs 34 SR)	Complete / massive tears; 12 months
Ruiz Ibán et al.^5^	RCT	124 (57 vs 57 analyzed)	Complete posterosuperior tears; ~25 months (2 years)
Chacón et al.^9^	RCT (double-blind)	60 (30 vs 30)	Small/medium (≤ 2.5 cm) fullthickness tears; 24 months
Wang et al.^9^	RCT	41	High-grade partial-thickness tears; 12 months

AR: augmented repair (bioinductive implant); SR: standard/conventional repair; RCT: randomized controlled trial.

**Table 2:** Quality of evidence (GRADE): bioinductive implant augmentation versus conventional repair.

Outcome	Studies (N)	Certainty of evidence
Re-tear (re-rupture)	4 RCTs (288)	Very low^1^,^2^,^3^
Function (functional scores)	4 RCTs (288)	Very low^1^,^2^,^3^

1 Risk of bias of the included studies;

2 Imprecision (small samples; wide confidence intervals);

3 Inconsistency/indirectness (heterogeneous tear types and follow-up periods). Note: GRADE (Grading of Recommendations Assessment, Development and Evaluation) is a tool used to assess and grade the certainty of evidence, based on five domains: risk of bias, imprecision, inconsistency, indirectness, and publication bias. The certainty of evidence is classified as very low, low, moderate, or high.

### Study Limitations

Future research should prioritize randomized, multicenter clinical trials with rigorous methodology, standardized lesion and patient profiles, and longer follow-up. Studies with this design are essential to more precisely define the long-term clinical and functional benefit of bioinductive augmentation relative to conventional repair, since the current evidence is limited and heterogeneous.

## CONCLUSION

There is very low-quality evidence that rotator cuff repair augmented with a bioinductive implant (patch) is more effective than conventional rotator cuff repair for the treatment of rotator cuff tears (including complete and massive tears), with respect to the outcomes of re-tear and function.

### Recommendation

Regarding the outcomes of re-tear and function, there is evidence, albeit of very low quality, that rotator cuff repair augmented with a bioinductive implant is superior to conventional repair for the treatment of rotator cuff tears.

Therefore, the Brazilian Society of Shoulder and Elbow Surgery (SBCOC) Consensus Project recommends that the surgeon individually assess patient- and disease-related characteristics to reach the best treatment decision. With respect to re-tear and function, this project suggests that, in selected cases, bioinductive implant augmentation may be preferred over conventional repair.

## Data Availability

The contents underlying the research are available in the manuscript.

## Appendix 1 Database search strategy.

**Table:**

**PUBMED**
#1 "Rotator Cuff Injuries"[Mesh] OR (Cuff Injury, Rotator) OR (Injury, Rotator Cuff) OR (Rotator Cuff Injury) OR (Rotator Cuff Tears) OR (Rotator Cuff Tear) OR (Tear, Rotator Cuff) OR (Tears, Rotator Cuff) OR (Rotator Cuff Tendinitis) OR (Rotator Cuff Tendinitides) OR (Tendinitis, Rotator Cuff) OR (Rotator Cuff Tendinosis) OR (Rotator Cuff Tendinoses) OR (Tendinoses, Rotator Cuff) OR (Tendinosis, Rotator Cuff) OR (Glenoid Labral Tears) OR (Glenoid Labral Tear) OR (Labral Tear, Glenoid) OR (Labral Tears, Glenoid) OR (Tear, Glenoid Labral)
#2 (bioinductive) OR (bioinductive patch) OR (bioinductive implant) OR (bioinductive collagen implant) OR (collagen patch) OR (collagen scaffold)
#3 #1 AND #2

**Table:**

**COCHRANE LIBRARY**
#1 (Rotator Cuff Injuries) OR (Cuff Injury, Rotator) OR (Injury, Rotator Cuff) OR (Rotator Cuff Injury) OR (Rotator Cuff Tears) OR (Rotator Cuff Tear) OR (Tear, Rotator Cuff) OR (Tears, Rotator Cuff) OR (Rotator Cuff Tendinitis) OR (Rotator Cuff Tendinitides) OR (Tendinitis, Rotator Cuff) OR (Rotator Cuff Tendinosis) OR (Rotator Cuff Tendinoses) OR (Tendinoses, Rotator Cuff) OR (Tendinosis, Rotator Cuff) OR (Glenoid Labral Tears) OR (Glenoid Labral Tear) OR (Labral Tear, Glenoid) OR (Labral Tears, Glenoid) OR (Tear, Glenoid Labral)
#2 (bioinductive) OR (bioinductive patch) OR (bioinductive implant) OR (bioinductive collagen implant) OR (collagen patch) OR (collagen scaffold)
#3 #1 AND #2

**Table:**

**EMBASE**
#1 ‘rotator cuff injury’/exp OR ‘cuff injury’ OR ‘cuff lesion’ OR ‘rotator cuff disease’ OR ‘rotator cuff disorder’ OR ‘rotator cuff disorders’ OR ‘rotator cuff injuries’ OR ‘rotator cuff injury’ OR ‘rotator cuff lesion’ OR ‘rotator cuff lesions’ OR ‘rotator cuff pathology’ OR ‘rotator cuff tendinopathies’ OR ‘rotator cuff tendinopathy’ OR ‘shoulder rotator cuff degeneration’ OR ‘shoulder rotator cuff lesion’
#2 (bioinductive) OR (bioinductive patch) OR (bioinductive implant) OR (bioinductive collagen implant) OR (collagen patch) OR (collagen scaffold)
#3 #1 AND #2

**Table:**

**REGIONAL PORTAL (BVS)**
#1 Mh:"Lesões do Manguito Rotador" OR (Lesões do Manguito Rotador) OR (Lesões do Ligamento Glenoidal) OR (Lesões do Ligamento Glenoídeo) OR (Lesões do Ligamento Glenóideo) OR (Roturas da Coifa dos Rotadores) OR (Roturas do Ligamento Glenoidal) OR (Roturas do Ligamento Glenoídeo) OR (Roturas do Ligamento Glenóideo) OR (Roturas do Manguito Rotador) OR (Ruptura do Manguito Rotador) OR (Rupturas da Coifa dos Rotadores) OR (Rupturas do Ligamento Glenoidal) OR (Rupturas do Ligamento Glenoídeo) OR (Rupturas do Ligamento Glenóideo) OR (Rupturas do Manguito Rotador) OR (Tendinite da Coifa dos Rotadores) OR (Tendinite do Manguito Rotador) OR (Tendinose da Coifa dos Rotadores) OR (Tendinose do Manguito Rotador) OR MH:C26.761.340$ OR MH:C26.803.063$ OR MH:C26.874.400$
#2 (bioinductive) OR (bioinductive patch) OR (bioinductive implant) OR (bioindutivo) OR (implante bioindutivo) OR (patch bioindutivo) OR (bioinductivo) OR (parche bioinductivo) OR (implante de colágeno)
#3 #1 AND #2

## References

[1] Kbibi MB, Verhaegen F, Debeer P (2024). The clinical efficacy of the Regeneten bioinductive implant in rotator cuff repair: a systematic review. Acta Orthop Belg.

[2] Warren JR, Domingo-Johnson ER, Sorensen AA, Cheng AL, Latz KH, Cil A (2024). Bioinductive patch as an augmentation for rotator cuff repair: a systematic review and meta-analysis. J Shoulder Elbow Surg.

[3] Hurley ET, Twomey-Kozack J, Doyle TR, Meyer LE, Meyer AM, Lorentz SG (2025). Bioinductive collagen implant has potential to improve rotator cuff healing: a systematic review. Arthroscopy.

[4] Longo UG, Marino M, de Sire A, Ruiz-Iban MA, D’Hooghe P (2025). The bioinductive collagen implant yields positive histological, clinical and MRI outcomes in the management of rotator cuff tears: a systematic review. Knee Surg Sports Traumatol Arthrosc.

[5] Ruiz Ibán MA, García Navlet M, Moros Marco S, Diaz Heredia J, Hernando A, Ruiz Díaz R (2025). Augmentation of a posterosuperior cuff repair with a bovine bioinductive collagen implant shows a lower retear rate but similar outcomes compared with no augmentation: 2-year results of a randomized controlled trial. Arthroscopy.

[6] McIntyre LF, Nherera L, Schlegel TF (2023). Resorbable bioinductive collagen implant is cost effective in the treatment of rotator cuff tears. Arthrosc Sports Med Rehabil.

[7] Alkhateeb J, Langley C, Wong I (2024). Arthroscopic augmentation of subscapularis revision repair using a bioinductive collagen implant. Arthrosc Tech.

[8] Ferreira de Barros AA (2023). Bioinductive scaffold augmentation in complete and massive rotator cuff tears: a prospective randomized trial. J Shoulder Elbow Surg.

[9] Chacón JAC, Rojo VR, Martin Martinez AM, Espierrez JC, Calvo VG, Calderón Meza JMC (2024). An isolated bioinductive repair vs sutured repair for full-thickness rotator cuff tears: 2-year results of a double-blinded, randomized controlled trial. J Shoulder Elbow Surg.

[10] Wang A, Breidahl W, Ek ET, Falconer T, D’Alessandro P, Ebert JR (2026). Early functional recovery is improved in patients treated with bioinductive collagen implant augmentation compared with standard arthroscopic repair of high-grade partial-thickness rotator cuff tears: a prospective randomized trial. Orthop J Sports Med.

[11] Morche J, Freitag S, Hoffmann F, Rissling O, Langer G, Nußbaumer-Streit B (2020). GRADE-Leitlinien: 18. Wie ROBINS-I und andere Instrumente zur Einschätzung des Risikos für Bias von nicht-randomisierten Studien verwendet werden sollten, um die Vertrauenswürdigkeit eines Evidenzkörpers zu bewerten [GRADE guidelines: 18. How ROBINS-I and other tools to assess risk of bias in nonrandomized studies should be used to rate the certainty of a body of evidence]. Z Evid Fortbild Qual Gesundhwes.

[12] Kaiser L, Hübscher M, Rissling O, Schulz S, Langer G, Meerpohl J (2021). GRADE-Leitlinien: 19. Bewertung der Vertrauenswürdigkeit der Evidenz für die Bedeutung von Endpunkten oder Werten und Präferenzen – Risiko für Bias und Indirektheit [GRADE Guidelines: 19. Assessing the certainty of evidence in the importance of outcomes or values and preferences: risk of bias and indirectness]. Z Evid Fortbild Qual Gesundhwes.

